# Clusterin ameliorates diabetic atherosclerosis by suppressing macrophage pyroptosis and activation

**DOI:** 10.3389/fphar.2025.1536132

**Published:** 2025-04-23

**Authors:** Lingling Xuan, Lulu Ren, Xiaoxu Kang, Rui Chang, Wen Zhang, Lili Gong, Lihong Liu

**Affiliations:** ^1^ Department of Pharmacy, Beijing Chao-Yang Hospital, Capital Medical University, Beijing, China; ^2^ Institute of Clinical Medical Sciences, China-Japan Friendship Hospital, Capital Medical University, Beijing, China; ^3^ Department of Pharmacy, China-Japan Friendship Hospital, Beijing, China

**Keywords:** clusterin, atherosclerosis, diabetes, macrophage pyroptosis, inflammation

## Abstract

**Background:**

It has been demonstrated that clusterin (CLU) is a protective protein involved in a variety of diseases and disorders. However, the role of CLU in diabetic atherosclerosis is not elucidative. The objective of this study is to investigate the role of CLU in diabetic atherosclerosis and the molecular mechanisms.

**Method:**

In *in vivo* experiments, *Clu* knockout and overexpressed murine models were used to investigate the role of Clu in diabetic atherosclerosis. Atherosclerotic plaque formation was determined by hematoxylin-eosin (H&E) staining and Oil Red O staining. F4/80 and CD68 levels were determined by immunohistochemical staining. Transmission electron microscopy was used to observe changes in cell pyroptosis morphology. NLRP3 and IL-1β levels were determined by Western blot and immunofluorescence staining. In *in vitro* experiments, TNF-α, IL-6, and IL-1β levels in THP-1 derived macrophages were determined by real-time qPCR and ELISA.

**Results:**

We found that *Clu*-overexpression reduced while *Clu* knockout promoted atherosclerotic plaque formation, macrophage infiltration and inflammatory factor expression in mouse aortic plaques. Consistently, *CLU* overexpression inhibits the production of TNF-α, IL-6, and IL-1β in THP-1 derived macrophages. Moreover, Clu inhibited the release of inflammatory factors and macrophage pyroptosis in diabetic atherosclerosis murine models.

**Conclusion:**

Our study revealed that CLU could ameliorate diabetic atherosclerosis via suppressing inflammatory factors release and pyroptosis of macrophage. CLU may be a promising therapeutic target for diabetic atherosclerosis.

## 1 Introduction

Diabetes is a metabolic disorder characterized by insulin resistance and dysfunction of beta cells, resulting in chronic hyperglycemia. Cardiovascular complications stand as the primary cause of mortality and morbidity in patients with diabetes (Karakasis et al., 2025; Joseph et al., 2022). Endothelial cell injury and dysfunction are considered to be the early key steps in the pathogenesis of atherosclerosis (Shi and Vanhoutte, 2017). Endothelial injury leads to inflammatory responses and endothelial dysfunction, resulting in damage to the endothelial integrity. Subsequently, monocytes aggregate and transform into macrophages that engulf lipids to form foam cells, leading to the formation of plaques and initiating the early lesions of atherosclerosis (Xu et al., 2021). Research has shown that macrophages represent the predominant immune cell population within atherosclerotic plaques, playing a pivotal role in the inflammatory response, intercellular communication, as well as the formation and rupture of plaques (Chen et al., 2022). Compared with atherosclerosis without diabetes, diabetes-associated atherosclerosis has more unstable plaques, with a higher risk of cardiovascular events resulting from plaque rupture (Li et al., 2023).

Pyroptosis is a programmed cell death mechanism. Multiple stimuli including NLRP3 inflammasome can activate the caspase family, which further cleaves the intracellular Gasdermin D protein (GSDMD) and interleukin (IL)-1β and IL-18 precursor into active forms. GSDMD can form pores on the cell membrane, leading to cell death and the release of intracellular inflammatory factors (Coll et al., 2022; Rao et al., 2022). Compared to apoptosis, pyroptosis occurs faster and is accompanied by the release of large amounts of pro-inflammatory factors (Rao et al., 2022). Pyroptosis has been found in vascular endothelial cells, macrophages and smooth muscle cells (He et al., 2021; Liu et al., 2023). Recent studies have shown that pyroptosis closely related to diabetic atherosclerosis and increases plaque instability (He et al., 2021; Zhu et al., 2022).

Clusterin (CLU), also known as Apolipoprotein J (ApoJ), is a molecular chaperone protein that belongs to the apolipoprotein family while also serving as a cellular protectant (Trougakos, 2013; Elias et al., 2022). It is involved in a variety of physiological and pathological processes, including cell proliferation, tissue regeneration and sperm maturation. In recent years, researchers have increasingly focused on the role of CLU in cardiovascular diseases (Park et al., 2014).

Low-density lipoprotein receptor-related protein 2 (LRP2), a member of the low-density lipoprotein superfamily, is widely distributed in various tissues and can bind to various ligands to participate in receptor-mediated endocytosis (Calvier et al., 2022; Beenken et al., 2023). LRP2 mediates the endocytosis and degradation of lipoproteins by binding to low density lipoprotein and very low-density lipoprotein. Research has shown that CLU is an important ligand for LRP2, and previous studies have found that CLU promotes the endocytosis and degradation of lipoproteins through LRP2-mediated endocytosis, as well as regulating appetite control in the hypothalamus (Gil et al., 2013; Bradley et al., 2019). Studies have shown that plasma CLU levels are independently associated with insulin resistance, indicating that it might be a potential biomarker for predicting metabolic disorders (Wittwer and Bradley, 2021; Wang et al., 2023). This suggests that CLU may play an important role in the development of diabetes, but the role and mechanism of CLU in diabetic atherosclerosis have not yet been elucidated. Therefore, our study aims to investigate the role of CLU in alleviating the development of diabetic atherosclerosis *in vitro* and *in vivo*, and to elucidate the molecular mechanism.

## 2 Materials and methods

### 2.1 Materials

Streptozotocin (STZ) and lipopolysaccharide (LPS) were purchased from Sigma Aldrich (St. Louis, MO, United States). Phorbol 12-myristate 13-acetate (PMA) was from Beyotime Biotechnology (Jiangsu, China). Oxidized low-density lipoprotein (ox-LDL) was from Beijing Bioynthesis Biotechnology Co., LTD (Beijing, China). The kits for determining total cholesterol (TC), low-density lipoprotein cholesterol (LDL-C), and triglyceride (TG) were purchased from Nanjing Jiancheng Institute of Biological Engineering (Nanjing, China). Anti-NLRP3 antibody was from Cell Signaling Technology (MA, United States). Anti-CD68, anti-F4/80, anti-Caspase-1, and anti-β-actin antibodies were from Proteintech Group (Wuhan, China). Anti-MAC2 antibody was from Servicebio (Wuhan, China). Anti-IL-1β antibody was from Abcam (Cambridge, UK). RPMI 1640 medium, and fetal bovine serum (FBS) were obtained from HyClone (Logan, UT, United States).

### 2.2 Subjects

Patients with diabetes and atherosclerosis (n = 19) and healthy controls (n = 32) were recruited from Beijing Chao-Yang Hospital. Diabetes and atherosclerosis were diagnosed by physicians. Patients were classified as having diabetes based on the following criteria: fasting blood glucose (FBG) ≥ 7.0 mmol/L, 2-h postprandial glucose (2-h PG) ≥ 11.1 mmol/L during an oral glucose tolerance test (OGTT), HbA1c ≥ 6.5%, or a known history of diabetes with current antidiabetic treatment (American Diabetes Association Professional Practice Committee, 2022). For the diabetes + atherosclerosis group, patients met the diabetes criteria and had clinical or imaging evidence of atherosclerosis, including the presence of atherosclerotic plaques or significant stenosis in major arteries confirmed by imaging modalities such as carotid ultrasound or coronary angiography (Gu et al., 2024). Exclusion criteria were as follows: (1) pregnant and lactating females; (2) diagnosis of cancer; (3) recent acute surgery operation; (4) acute myocardial infarction and/or acute cerebral infarction in the past 4 weeks; (5) psychiatric disorder. This study was approved by the Research Ethics Committee of Beijing Chao-Yang Hospital (No. 2020-ke416). We used residual blood samples remaining from routine clinical biochemistry testing; therefore, informed consent was waived for this study. Blood samples were collected and centrifuged at 3,000 g for 10 min at room temperature. The supernatants were collected for CLU quantification by ELISA (Raybiotech, Norcross, GA, United States).

### 2.3 Mice

Specific pathogen free male C57BL/6 mice and *ApoE* ^−/−^ mice (6 weeks) were purchased from Beijing HFK Bioscience Co., Ltd. (Beijing, China). *Clu* knockout (*Clu* ^*−/−*^) on C57BL/6 genetic background (male and female, 6–8 weeks) were constructed using CRISPR technology by Cyagen Bioscience, Inc. (Guangzhou, China). All the mice were kept under a 12 h light-dark cycle at 20°C–26°C with free access to food and water.

The adeno-associated virus (AAV) used in this study was constructed as previously reported (Ren et al., 2019). To investigate the effects of *Clu* overexpression on diabetic atherosclerosis development, C57BL/6 mice and *ApoE* ^−/−^ mice were divided into 5 groups (n = 8): C57BL/6 + normal diet (ND) + AAC-NC (Negative control), *ApoE* ^−/−^ + high fat diet (HFD) + AAV-NC (atherosclerosis + AAV-NC), *ApoE* ^−/−^ + HFD + AAV-*Clu* (atherosclerosis + AAV-*Clu*), *ApoE* ^−/−^ + HFD + STZ + AAV-NC (diabetic atherosclerosis + AAV-NC), and *ApoE* ^−/−^ + HFD + STZ + AAV-*Clu* (diabetic atherosclerosis + AAV-*Clu*). C57BL/6 mice were fed with ND and the *ApoE* ^−/−^ mice were fed with HFD (D12108C) during the entire experimental period. On weeks 1 and 4, the mice received a tail vein injection of AAV-NC or AAV-*Clu* at a dose of 10^11^ PFU. On week 4, 24 h after AAV injection, mice in the *ApoE* ^−/−^ + HFD + STZ + AAV-*Clu* and *ApoE* ^−/−^ + HFD + STZ + AAV-NC groups were intraperitoneal injected with STZ at the dose of 35 mg/kg for 5 consecutive days. Mice in the C57BL/6 + ND + AAC-NC, *ApoE* ^−/−^ + HFD + AAV-*Clu,* and *ApoE* ^−/−^ + HFD + AAV-NC groups were injected with sodium citrate buffer. On week 16, the mice were euthanized under anesthesia, and their plasma and aortas were collected for further experiments. Body weight was measured at the end of the experiment. FBG was measured by blood glucose meter. Serum lipids levels including TC, LDL, and TG were measured by enzymatic assay according to the manufacture’s introduction. Clu levels in serum was determined by ELISA (Raybiotech, Norcross, GA, United States).

*Clu* ^−/−^ mice were crossed with *ApoE* ^−/−^ mice to derive *ApoE* ^−/−^ *Clu* ^−/−^ mice. To investigate the effects of *Clu* knockout on diabetic atherosclerosis development, C57BL/6 mice, *ApoE* ^−/−^ mice and *ApoE* ^−/−^ *Clu* ^−/−^ mice were divided into 4 groups (n = 8): C57BL/6 + ND group, *ApoE* ^−/−^ + HFD group, *ApoE* ^−/−^ + HFD + STZ group, and *ApoE* ^−/−^ *Clu* ^−/−^ + HFD + STZ group. C57BL/6 mice were fed with ND, and the *ApoE* ^−/−^ and *ApoE* ^−/−^ *Clu* ^−/−^ mice were fed with HFD (D12108C) during the entire experimental period. On week 4, mice in the *ApoE* ^−/−^ + HFD + STZ group and the *ApoE* ^−/−^ *Clu* ^−/−^ + HFD + STZ group were intraperitoneal injected with STZ as described above. On week 16, the mice were euthanized. The body weight, FBG, and serum lipid levels were measured as described above.

### 2.4 Oil Red O staining

For Oil Red O staining of the whole aortas, fresh isolated whole aortas were dissected under a stereomicroscope and fixed in 4% paraformaldehyde for 24 h. The vessel wall was unraveled along the lumen and stained with Oil Red O. Rinse in 60% isopropyl alcohol and then in water. The images were acquired using stereomicroscopy.

For quantification lipids accumulation and atherosclerotic lesions in the aortic root, aortas samples were sectioned into frozen tissue slices. The sections were incubated with 1% Oil Red O solution for 10 min at room temperature, followed by rinsing in 60% isopropanol and water. Then the nuclei were stained with hematoxylin solution. The sections were examined using an optical microscope.

### 2.5 Hematoxylin-eosin staining

After fixation in 4% paraformaldehyde for 48 h, the aortic sinus was embedded in paraffin and sectioned into 4 μm slices, and then stained with hematoxylin and eosin. The stained sections were examined using an optical microscope.

### 2.6 Immumohistochemistry

Paraffin-embedded tissue sections were deparaffinized and rehydrated. Then the antigen retrieval was performed. Nonspecific binding was blocked with serum. Sections were incubated overnight with the primary antibody (CD68 and F4/80) at 4°C, followed by washing and incubation with a secondary antibody. Detection was performed using DAB. Counterstaining was done with hematoxylin, dehydration with ethanol, and mounting with a coverslip. Images were captured using an optical microscope.

### 2.7 Immunofluorescence staining

Paraffin-embedded tissue sections were deparaffinized and rehydrated. Antigen retrieval was performed using citrate buffer. Nonspecific binding was blocked with 10% serum for 1 h. Sections were incubated overnight at 4°C with a mixture of primary antibodies (F4/80, MAC2, Clu, and NLRP3). After washing with PBS, sections were incubated with fluorescently labeled secondary antibodies for 1 h at room temperature. Nuclei were counterstained with DAPI for 10 min. Autofluorescence was quenched by treating the tissue sections with autofluorescence quencher. Slides were washed with PBS and mounted with antifade mounting medium. Fluorescent images were captured using a fluorescence microscope.

### 2.8 Determination of inflammatory cytokines

Aortas were first homogenized in TRIzol reagent, and total RNA was extracted following the manufacturer’s protocol. RNA quality and quantity were assessed using a NanoDrop 2000 (Thermo Fisher Scientific, United States). cDNA was synthesized from 1 μg of total RNA using a reverse transcription kit. Real-time quantitative PCR (RT-qPCR) was performed using SYBR Green Master Mix. The 2^−ΔΔCt^ method was applied. TNF-α, IL-6, and IL-1β levels in serum was determined by ELISA according to the manufacture’s introduction (BioLegend, San Diego, CA, United States).

The adenoviral virus used in this study was constructed as previously reported (Ren et al., 2019). THP-1 cells were incubated with 300 nM PMA for 48 h and then washed with PBS for 2 times. Subsequently, the cells were infected with adenovirus expressing *CLU* (Ad-*CLU*) or negative control adenovirus (Ad-NC) for 48 h. The cells were then stimulated with 1 mg/L LPS or 75 μg/mL ox-LDL for 24 h. The mRNA levels of TNF-α, IL-6, and IL-1β was determined by RT-qPCR as described above. Primers were shown in Supplementary Table 1. The protein levels of TNF-α, IL-6, and IL-1β in supernatant were determined by ELISA according to the manufacture’s introduction (BioLegend, San Diego, CA, United States).

### 2.9 Transmission electron microscopy

Aortas were carefully excised and immediately placed in a fixative solution containing 2.5% glutaraldehyde and 2% paraformaldehyde in 0.1 M phosphate buffer (pH 7.4) for 2 h at room temperature. The tissue was then washed with 0.1 M phosphate buffer and post-fixed in 1% osmium tetroxide for 1 h. Following dehydration through a graded series of ethanol solutions and propylene oxide, the tissue was infiltrated with epoxy resin and embedded. Ultrathin sections were cut. Sections were stained with uranyl acetate and lead citrate for contrast enhancement. Specimens were examined under a transmission electron microscope.

### 2.10 Western blotting

Aortas were homogenized in ice-cold RIPA buffer supplemented with protease inhibitors. Protein concentration was determined using the BCA Assay Kit (Beyotime, China). Protein were separated by SDS-PAGE and then transferred to a PVDF membrane. The membrane was then blocked with 5% milk for 2 h at room temperature. Membranes were incubated overnight at 4°C with primary antibodies (NLRP3, IL-1β, Caspase-1, and β-actin). After washing, membranes were incubated with fluorescently labeled secondary antibodies. Protein bands were visualized using an LICOR Odyssey scanner (LI-COR, United States).

### 2.11 Statistical analysis

Variables with a normal distribution were summarized as means ± SEM. Variables without a normal distribution were reported as median (interquartile range). The comparison between 2 groups was conducted using a t-test. Nonparametric tests were used for analyzing data that were not normally distributed. Means across 3 groups or more were compared using one-way ANOVA. Sex differences were compared using chi-square test. Statistical significance was set at *P* < 0.05.

## 3 Results

### 3.1 *Clu* overexpression reduces atherosclerotic plaque formation in diabetic atherosclerosis murine models

To investigate the role of CLU in diabetic atherosclerosis, a total of 19 individuals with diabetes and atherosclerosis, and 32 healthy controls were enrolled in the study. The clinical characteristics of the participants were shown in Supplementary Table 2. Compared to healthy controls, diabetic atherosclerosis patients had lower levels of TC, LDL-C, and high-density lipoprotein cholesterol (HDL-C). FBG levels did not significantly differ between diabetic atherosclerosis patients and healthy controls, possibly due to the use of antidiabetic medications. We measured serum CLU levels by ELISA (Supplementary Figure S1A). Compared to healthy controls, patients with diabetes and atherosclerosis showed a significant elevation in serum CLU levels (0.93 ± 0.04 mg/mL vs. 0.79 ± 0.03 mg/mL, *P* = 0.008). Next, we verified the expression of CLU levels using GEO dataset (GSE221615). Compared to patients with atherosclerosis alone, diabetic atherosclerosis patients had higher mRNA levels of CLU (Supplementary Figures S1B, C).

**FIGURE 1:**
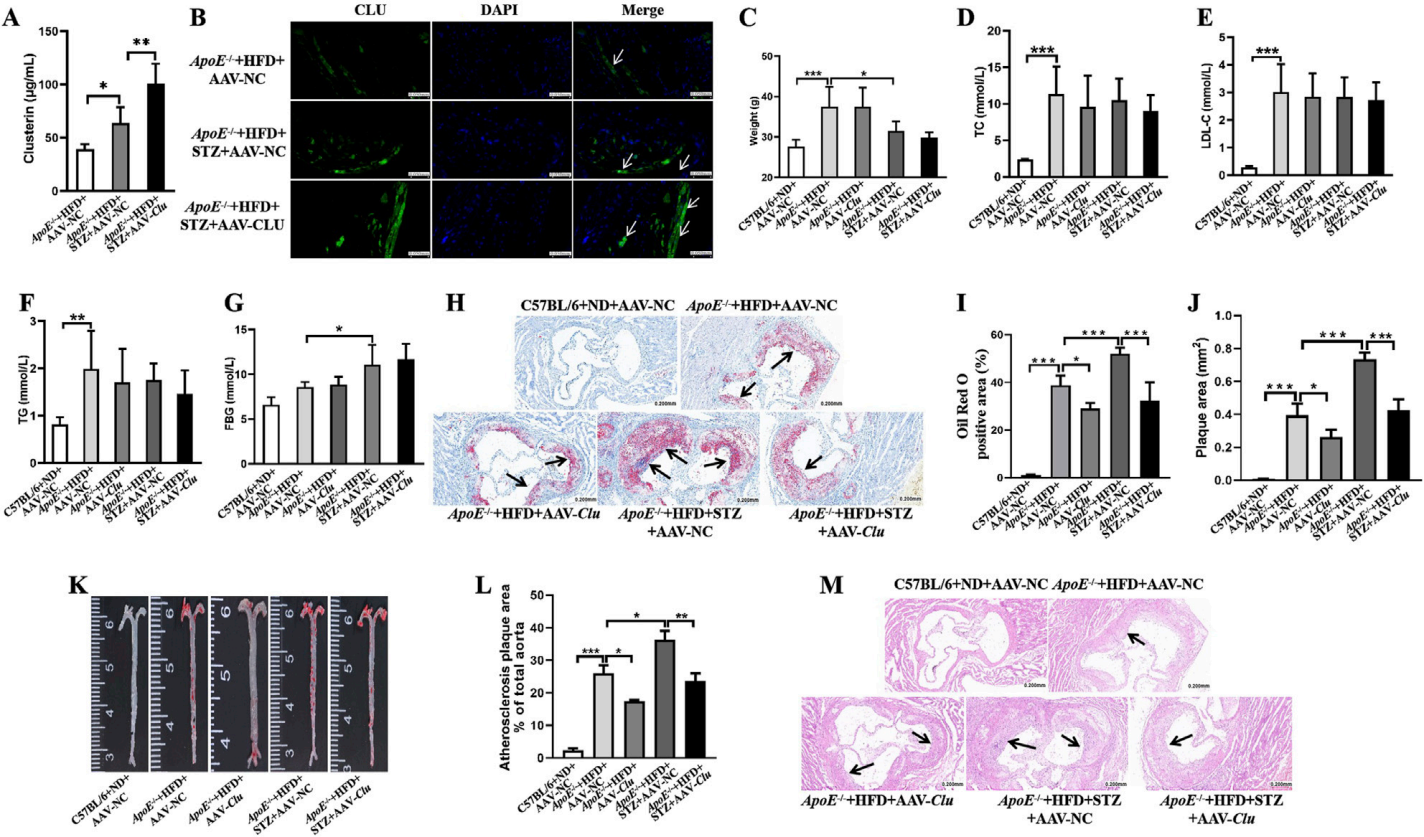
*Clu* overexpression reduces atherosclerotic plaque formation in diabetic atherosclerosis murine models. **(A)** Clu levels in serum. **(B)** Representative images of immunofluorescence staining for Clu in atherosclerotic lesions. Scale bar: 0.050 mm. **(C)** Body weight. **(D)** TC levels in serum. **(E)** LDL-C levels in serum. **(F)** TG levels in serum. **(G)** FBG levels. **(H)** Representative images of Oil red O staining in aortic sinuses area. Scale bar: 0.200 mm. **(I)** Quantiffcation of the Oil Red O positive area. **(J)** Quantiffcation of plaque area. **(K)** Representative images of whole aortas stained with Oil Red O. **(L)** Areas of the aortic lesion were expressed as percentage of aorta areas. **(M)** H&E-stained aortic root lesions. Scale bar: 0.200 mm n = 4 per group. *P < 0.05, **P < 0.01, ***P < 0.001.

To further investigate the role of Clu in diabetic atherosclerosis, we established the diabetic atherosclerosis murine model using *ApoE* ^−/−^ mice by administering a HFD combined with low-dose multiple intraperitoneal injections of STZ. Additionally, we included a non-diabetic control group fed a HFD without STZ to evaluate the effects of *Clu* overexpression in the absence of diabetes. The mice were injected with adeno-associated virus that overexpresses *Clu* (AAV-*Clu*) to observe the impact of *Clu* overexpression on the development and progression of non-diabetic and diabetic atherosclerosis. The detection of Clu levels in serum by ELISA and Immunofluorescence staining of the atherosclerotic plaques revealed that compared with *ApoE* ^−/−^ + HFD + AAV-NC mice, Clu levels in serum (Figure 1A) and atherosclerotic plaques (Figure 1B) were moderately increased in *ApoE* ^−/−^ + HFD + STZ + AAV-NC mice. However, in the *ApoE* ^−/−^ + HFD + STZ + AAV-*Clu* group, Clu levels in serum and atherosclerotic plaques were significantly higher, indicating successful overexpression of *ApoE* ^−/−^ in the target tissues (Figure 1B). *Clu* overexpression did not significantly alter body weight, TC, LDL-C, TG, or FBG in mice (Figures 1C–G).

We then evaluated the atherosclerosis plaque formation by Oil Red O staining and H&E staining. Compared with the mice in the *ApoE* ^−/−^ + HFD + AAV-NC group, a significant increase in Oil Red O positive area and arterial plaque area was observed in the *ApoE* ^−/−^ mice injected with STZ (*ApoE* ^*−/−*^ + HFD + STZ + AAV-NC), highlighting the exacerbating effect of diabetes on atherosclerosis. In the non-diabetic group overexpressing *Clu* (*ApoE* ^*−/−*^ + HFD + AAV-*Clu*), we observed a significant reduction in both the lipid content and overall plaque area, as evidenced by decreased Oil Red O staining area and intensity, as well as reduced plaque size in H&E staining (Figures 1H–M). This suggests that *Clu* overexpression can mitigate atherosclerosis progression even in the absence of diabetes.

In the diabetic group (*ApoE* ^*−/−*^ + HFD + STZ + AAV-*Clu*), the protective effects of *Clu* overexpression were even more pronounced. Mice overexpressing *Clu* exhibited a notable reduction in lipid content within the plaque, as evidenced by decreased Oil Red O staining area and intensity compared to the diabetic control group (*ApoE* ^*−/−*^ + HFD + STZ + AAV-NC). Additionally, H&E staining revealed a significant decrease in overall plaque area, indicating that *Clu* overexpression can effectively mitigate the exacerbating effect of diabetes on atherosclerosis (Figures 1H–M).

These findings indicated that overexpression of *Clu* significantly decreased atherosclerotic plaque formation and lipid accumulation in both diabetic and non-diabetic *ApoE* ^*−/−*^ mice fed a HFD. However, the protective effects of Clu were more pronounced in the diabetic atherosclerosis model. Moreover, diabetic atherosclerosis is more severe and difficult to manage. Therefore, subsequent experiments were primarily focused on elucidating the mechanisms underlying Clu’s protective effects in diabetic atherosclerosis.

### 3.2 *Clu* konckout promotes atherosclerotic plaque formation in diabetic atherosclerosis murine models

**FIGURE 2:**
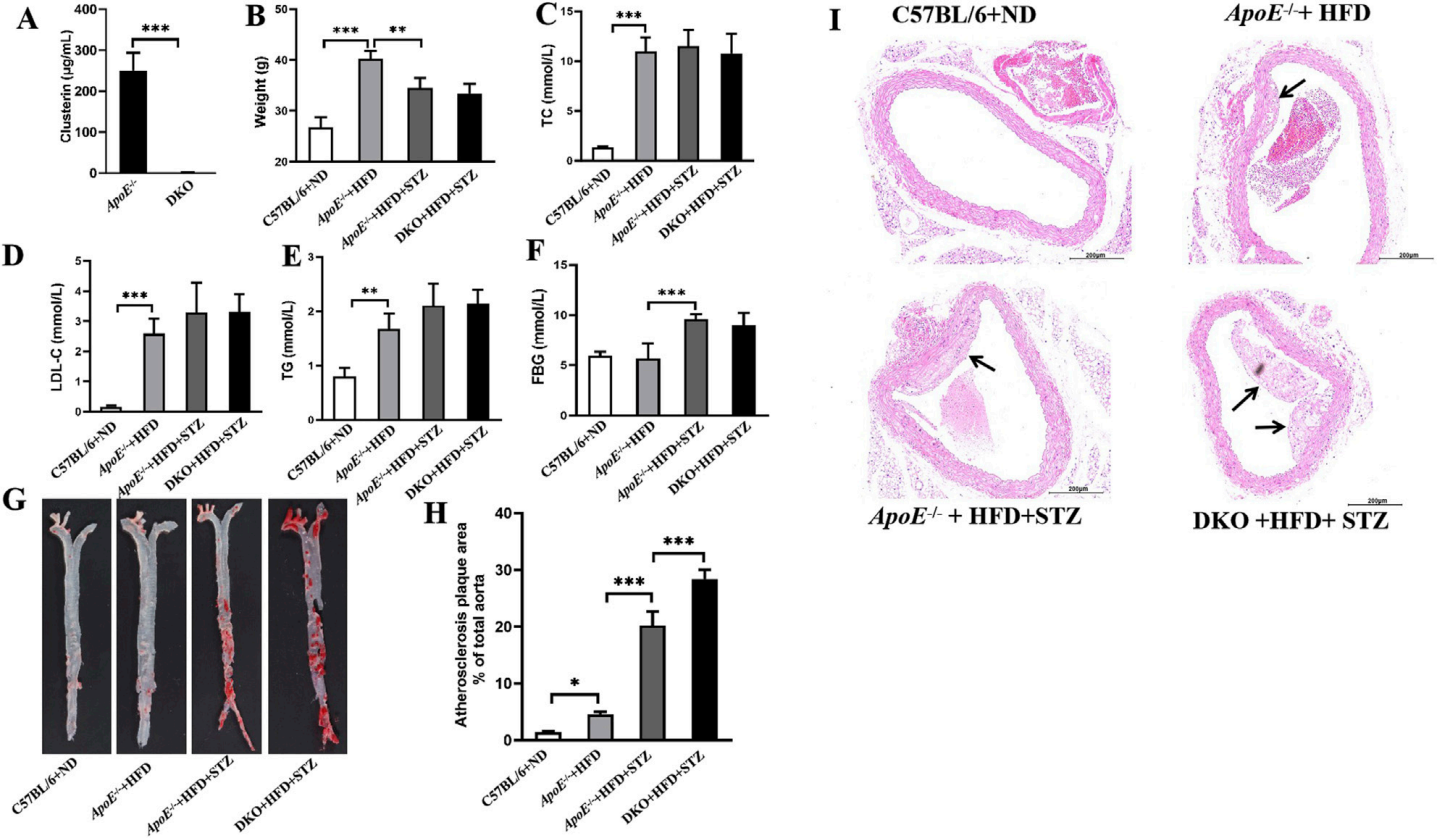
*Clu* knockout promotes atherosclerotic plaque formation in diabetic atherosclerosis murine models. **(A)** Clu levels in serum. **(B)** Body weight. **(C)** TC levels in serum. **(D)** LDL-C levels in serum. **(E)** TG levels in serum. **(F)** FBG levels. **(G)** Representative images of whole aortas stained with Oil Red O. **(H)** Areas of the aortic lesion were expressed as percentage of aorta areas. **(I)** H&E-stained aortic root lesions. Scale bar: 0.200 mm. DKO: *ApoE* ^−/−^ *Clu* ^−/−^ mice. n = 4 per group. *P < 0.05, **P < 0.01, ***P < 0.001.

Given the above research results showed that *Clu* overexpression could alleviate the development of diabetic atherosclerosis, we further established *Clu* knockout mice (*Clu* ^−/−^) to observe whether *Clu* knockout can promote the development of diabetic atherosclerosis, thereby determining the role of Clu in the occurrence and progression of diabetic atherosclerosis. Systemic *Clu* knockout mice (*Clu* ^−/−^) were crossed with *ApoE* ^−/−^ mice to generate *ApoE* ^−/−^ *Clu* ^−/−^ mice. To confirm the successful knockout of the *Clu* gene, we measured serum Clu levels in both *ApoE* ^−/−^ and *ApoE* ^−/−^ *Clu* ^−/−^ mice. As expected, Clu was virtually undetectable in the serum of *ApoE* ^−/−^ *Clu* ^−/−^ mice, confirming the successful establishment of the *Clu* knockout model (Figure 2A). In line with the aforementioned results, there was no significant difference in body weight, FBG, and the serum levels of TC, LDL-C, and TG between *ApoE* ^−/−^ *Clu* ^−/−^ mice and their littermate *ApoE* ^−/−^ controls (Figures 2B–F).

Oil Red O staining and H&E staining were also used to evaluate the atherosclerotic plaque formation. As shown in Figures 2G, H, *Clu* knockout significantly increased the aortic plaque area. Moreover, *Clu* knockout lead to a notable increase in atherosclerotic pathological plaque in aortic root (Figure 2I). Together, these results demonstrated the critical role of Clu in diabetic atherosclerosis.

### 3.3 *Clu* overexpression inhibits while *Clu* knockout enhances macrophage infiltration and inflammatory factor expression in mouse aortic plaques

**FIGURE 3:**
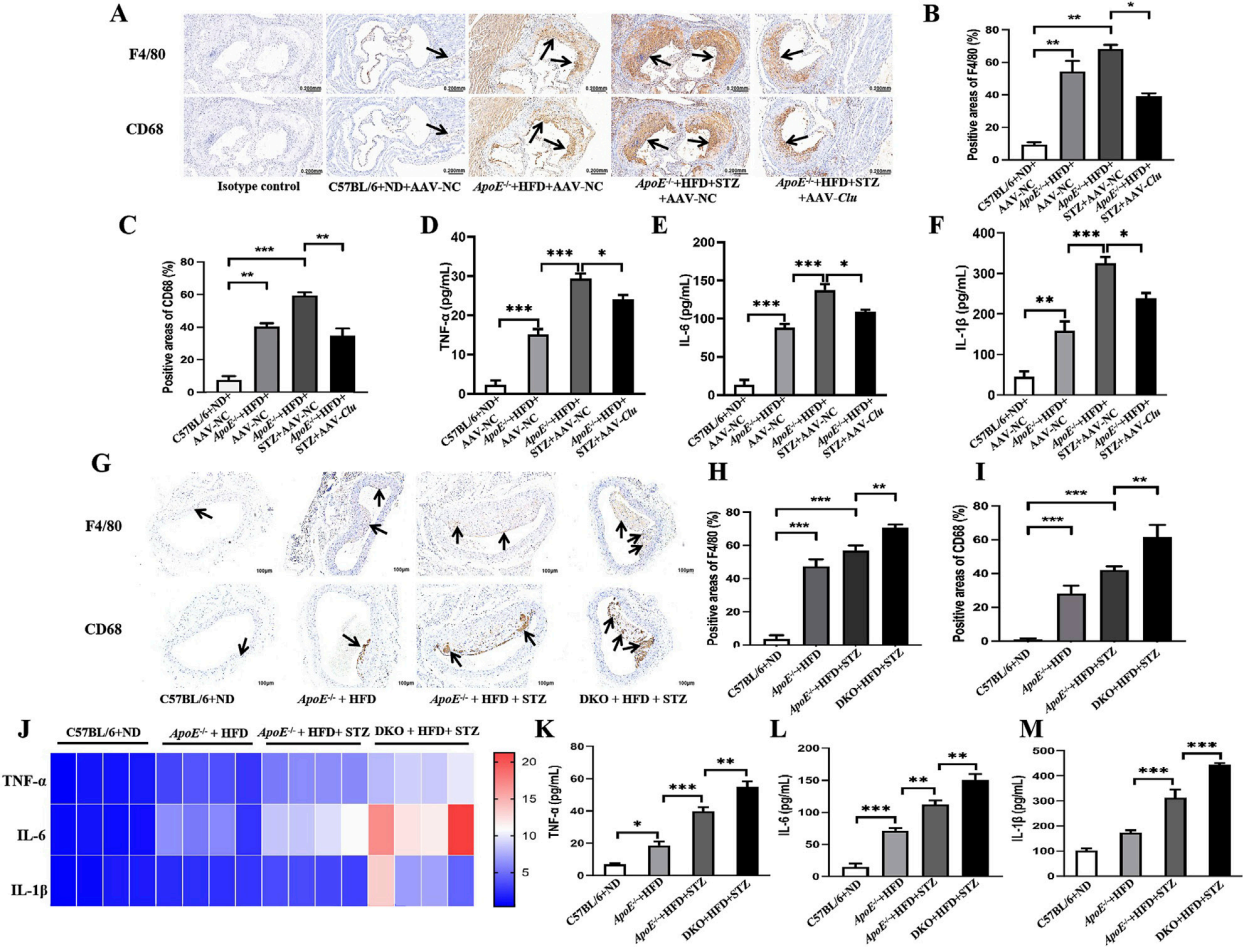
Effects of Clu on macrophage infiltration and inflammatory factor expression in mouse aortic plaques. **(A)** Representative images of immunohistochemical staining for F4/80 and CD68 in atherosclerotic lesions from mice injected with AAV-NC or AAV-*Clu*. Scale bar: 0.200 mm. **(B)** Quantitative data of immunohistochemical staining of F4/80. **(C)** Quantitative data of immunohistochemical staining of CD68. **(D–F)** TNF-α, IL-6, and IL-1β levels in serum determined by ELISA. **(G)** Representative images of immunohistochemical staining for F4/80 and CD68 in atherosclerotic lesions of C57BL/6, *ApoE* ^−/−^ and *ApoE* ^−/−^ *Clu* ^−/−^ mice. Scale bar: 0.200 mm. **(H)** Quantitative data of immunohistochemical staining of F4/80. **(I)** Quantitative data of immunohistochemical staining of CD68. **(J)** TNF-α, IL-6, and IL-1β levels in aortas determined by RT-qPCR. **(K–M)** TNF-α, IL-6, and IL-1β levels in serum determined by ELISA. DKO: *ApoE* ^−/−^ *Clu* ^−/−^ mice. n = 4 per group. *P < 0.05, **P < 0.01, ***P < 0.001.

Macrophages play a central role in plaque formation (Katakami, 2018). In the early stages of atherosclerosis, monocytes migrate into the subendothelial layer of the intima where they differentiate into macrophages. Macrophages phagocytose accumulated lipoproteins, forming cholesterol-rich foam cells, which promote plaque formation. Furthermore, macrophages can produce various pro-atherosclerotic cytokines and chemokines. As shown in Figures 3A–C, compared with the mice in the *ApoE* ^−/−^ + HFD + AAV-NC group, the expression of the monocyte/macrophage biomarkers CD68 and F4/80 was significantly increased in *ApoE* ^−/−^ mice injected with STZ (*ApoE* ^−/−^ + HFD + STZ + AAV-NC). However, the elevated expression of CD68 and F4/80 was significantly reduced by *Clu* overexpression. Additionally, the levels of pro-inflammatory cytokines TNF-α, IL-6, and IL-1β were significantly decreased in serum of mice overexpressing *Clu* compared to those in the *ApoE* ^*−/−*^ + HFD + STZ + AAV-NC group (Figures 3D–F). These findings indicated that *Clu* overexpression effectively attenuates macrophage infiltration and inflammatory factor expression.

Next, we assessed the effects of *Clu* knockout on macrophage infiltration and inflammatory factor expression. As expected, the expression of the monocyte/macrophage biomarkers CD68 and F4/80 was significantly increased by *Clu* knockout (Figures 3G–I). Moreover, compared with their littermate *ApoE* ^−/−^ controls, the *ApoE* ^−/−^ *Clu* ^−/−^ mice exhibited higher levels of TNF-α, IL-6, and IL-1β in both the aortas (Figure 3J) and serum (Figures 3K–M). These results indicated that Clu could modulate macrophage infiltration and inflammatory factor expression in mouse aortic plaques.

### 3.4 *CLU* overexpression inhibits the production of macrophage inflammatory cytokines *in vitro*

**FIGURE 4:**
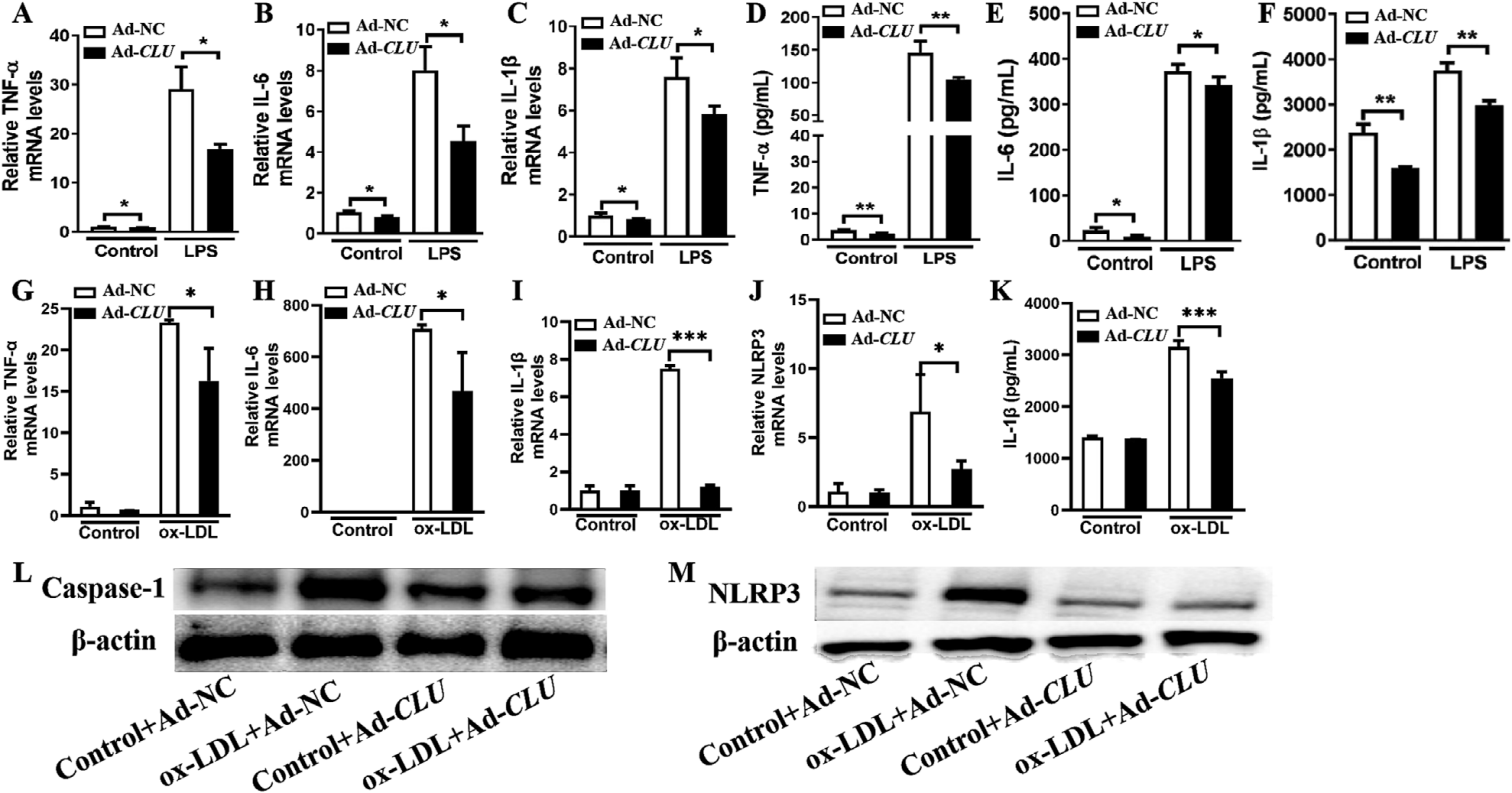
*CLU* overexpression suppresses the production of inflammatory cytokines in THP-1-derived macrophages. THP-1 cells were treated with PMA for 48 h and subsequently infected with Ad-NC or Ad-*CLU* for an additional 48 h. The cells were then stimulated with LPS, ox-LDL or vehicle for 24 h. **(A–C)** TNF-α, IL-6, and IL-1β levels in LPS-stimulated macrophages determined by RT-qPCR. **(D–F)** TNF-α, IL-6, and IL-1β levels in LPS-stimulated macrophages determined by ELISA. **(G–J)** TNF-α, IL-6, IL-1β, and NLRP3 levels in ox-LDL-stimulated macrophages determined by RT-qPCR. **(K)** IL-1β levels in ox-LDL-stimulated macrophages determined by ELISA. **(L–M)** Caspase-1 and NLRP3 levels in ox-LDL-stimulated macrophages determined by Western blotting. n = 3 per group. *P < 0.05, **P < 0.01, ***P < 0.001.

To further confirm the role of CLU in inhibiting macrophage activation, we investigated the effect of *CLU* overexpression on the secretion of inflammatory factors in macrophages *in vitro*. THP-1 cells were differentiated into macrophages by PMA stimulation and then stimulated with LPS or ox-LDL. Compared with the cells infected with negative control adenovirus (Ad-NC), *CLU* overexpression significantly reduced the TNF-α, IL-6, and IL-1β mRNA levels in macrophages stimulated by LPS (Figures 4A–C). Specifically, in LPS-stimulated macrophages, *CLU* overexpression led to a notable reduction in the protein levels of TNF-α, IL-6, and IL-1β, with average values decreasing from 145.20 ± 9.04 pg/mL, 374.20 ± 8.10 pg/mL, and 3,756.00 ± 99.89 pg/mL to 104.40 ± 1.44 pg/mL (*P* < 0.001), 342.50 ± 10.43 pg/mL (*P* = 0.049), and 2,985.00 ± 57.36 pg/mL (*P* < 0.001), respectively (Figures 4D–F). In addition, when macrophages were stimulated with ox-LDL, *CLU* overexpression significantly reduced the mRNA levels of TNF-α, IL-6, IL-1β, and NLRP3 (Figures 4G–J). The protein level of IL-1β was also significantly lowered by *CLU* overexpression, decreasing from 3,157.31 ± 69.33 pg/mL to 2551.40 ± 70.87 pg/mL (*P* < 0.05) (Figure 4K). Furthermore, Western blotting analysis demonstrated that *CLU* overexpression significantly decreased the protein levels of NLRP3 and caspase-1 in ox-LDL-stimulated macrophages (Figures 4L, M). These results indicated that CLU overexpression effectively inhibited the activation of the NLRP3 inflammasome and the production of pro-inflammatory cytokines in response to both LPS and ox-LDL stimulation.

### 3.5 CLU suppress macrophages pyroptosis

**FIGURE 5:**
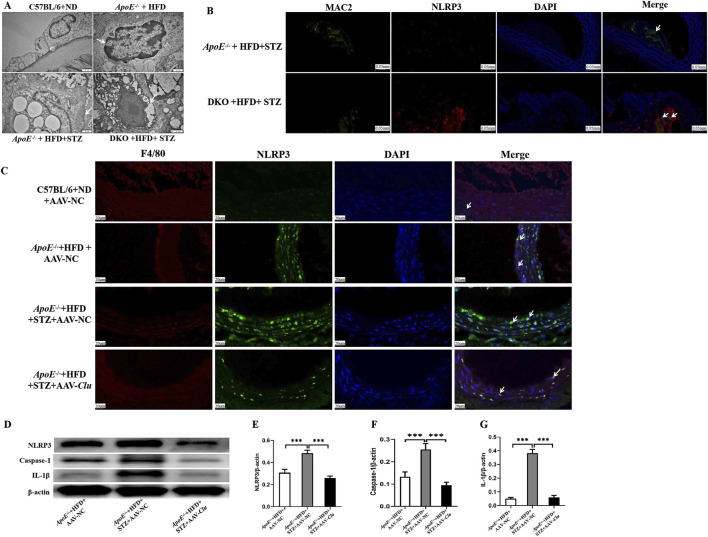
Clu suppressed macrophages pyroptosis *in vivo*. **(A)** Representative transmission electron microscopy images of atherosclerotic lesions in *ApoE* ^−/−^ and *ApoE* ^−/−^ *Clu* ^−/−^ mice. Scale bar: 1 μm. **(B)** Representative images of immunofluorescence staining for MAC2 and NLRP3 in atherosclerotic lesions of *ApoE* ^−/−^ and *ApoE* ^−/−^ *Clu* ^−/−^. Scale bar: 0.050 mm. **(C)** Representative images of immunofluorescence staining for F4/80 and NLRP3 in atherosclerotic lesions of mice injected with AAV-NC or AAV-*Clu*. Scale bar: 20 μm. **(D)** Western blotting for NLRP3, caspase-1, and IL-1β in mice injected with AAV-NC or AAV-*Clu*. **(E)** Relative quantification of the NLRP3 expression. **(F)** Relative quantification of the caspase-1 expression. **(G)** Relative quantification of the IL-1β expression. DKO, *ApoE* ^−/−^ *Clu* ^−/−^. n = 4 per group. ***P < 0.001.

Growing evidence highlights the pivotal role of macrophages pyroptosis in the progression and instability of atherosclerotic plaques. Pyroptosis not only causes local inflammation but also amplifies the inflammatory response and aggravates plaque instability (Wei et al., 2023). NLRP3 activation causes the activation of caspase-1, which cleaves pro-IL-1β to the mature form IL-1β and induces cell pyroptosis. Our transmission electron microscopy revealed that in *ApoE* ^−/−^ *Clu* ^−/−^ mice, the nuclear envelope became irregular, the nucleus displayed a condensed appearance and shrank in size, and vesicles were observed in the cytoplasm surrounding the nucleus (Figure 5A). These ultrastructural changes are characteristic hallmarks of pyroptosis, suggesting that *Clu* knockout exacerbates this form of cell death in the vasculature.

To further investigate the impact of Clu on macrophage pyroptosis, we performed dual immunofluorescence staining for NLRP3 and MAC2 in the aortic tissues of *ApoE* ^*−/−*^ + HFD + STZ mice and *ApoE* ^−/−^ *Clu* ^−/−^ + HFD + STZ. The results showed that NLRP3 expression was significantly upregulated in macrophages (identified by MAC2 staining) in the vessels of *ApoE* ^−/−^ *Clu* ^−/−^ mice compared with *ApoE* ^*−/−*^ controls (Figure 5B). Together with the transmission electron microscopy findings, these results suggest that *Clu* knockout promotes NLRP3-mediated pyroptosis specifically in macrophages, potentially contributing to increased inflammation and plaque instability.

In addition, we investigated the effect of *Clu* overexpression on NLRP3 and IL-1β expression in atherosclerotic plaques. Mice were administered AAV-*Clu* or AAV-NC (negative control) to modulate Clu expression. Immunofluorescence staining revealed that the expression levels of NLRP3 were lower in macrophages of the aortic plaques from mice in the *ApoE* ^−/−^ + HFD + STZ + AAV-*Clu* group compared with those in the *ApoE* ^−/−^ + HFD + STZ + AAV-NC group (Figure 5C). This demonstrated that while *Clu* deficiency promoted NLRP3 expression, *Clu* overexpression effectively inhibited it, highlighting the regulatory role of Clu in modulating NLRP3 and pyroptosis. Additionally, Western blotting analysis revealed that *Clu* overexpression led to decreased expression levels of NLRP3, Caspase-1, and IL-1β in these plaques (Figures 5D–G). These results collectively highlight the essential role of Clu in regulating macrophage pyroptosis.

## 4 Discussion

Accumulating evidence suggests that inhibition of macrophage inflammatory responses and pyroptosis could attenuate atherosclerosis and cardiovascular complications in diabetes (Josefs et al., 2020; Wang et al., 2024). In our study, we used gene knockout and overexpressed mouse models to investigate the role of Clu in diabetic atherosclerosis and its molecular mechanisms. We found that Clu could inhibit plaque formation and increase the stability of plaques in diabetic atherosclerosis mice, which is of great significance for preventing cardiovascular events. Moreover, our data indicated that Clu could inhibit the release of inflammatory factors and macrophage pyroptosis. Several studies have shown that Clu has immune-regulatory functions (Hong et al., 2016; De Miguel et al., 2021; Ren et al., 2023; Yang et al., 2023). Tony Wyss-Coray et al. reported that Clu could reduce neuroinflammatory gene expression in mice with acute brain inflammation and Alzheimer’s disease (De Miguel et al., 2021). Also, it’s suggested that Clu regulates allergic airway inflammation by diminishing CCL20-mediated recruitment of dendritic cells (Hong et al., 2016). Our findings elucidate a novel role for Clu in the immune regulation. Moreover, our data highlight the protective function of Clu. In our study, we observed that the serum level of CLU was upregulated in diabetic atherosclerosis patients within our cohort, as well as in the GEO dataset of diabetic atherosclerosis patients and in our diabetic atherosclerosis models, which is similar with small heat shock proteins (sHSPs) as a stress-induced protein (Wyatt et al., 2009). Moreover, our findings indicate that overexpression of Clu inhibits the development of diabetic atherosclerosis, while CLU knockout promotes its progression. These results collectively affirm the protective role of CLU in diabetic atherosclerosis. Prior research has established that CLU functions as a stress-responsive protein, mitigating oxidative stress and apoptosis under various stress conditions (Carnevali et al., 2006; Kang et al., 2013; Ren et al., 2019). Our data highlight an additional protective function of CLU.

Macrophages promote the formation of atherosclerosis by engulfing lipids to form foam cells (Chuang et al., 2024). In diabetes, the hyperglycemia condition facilitates the inflammatory response of macrophages and the formation of foam cells (Kaplan et al., 2012). Therefore, inhibiting the inflammatory response of macrophages in diabetes can effectively mitigate vascular damage and plaque formation. Many drugs target macrophages to inhibit plaque formation, such as the new cholesterol-lowering drug ezetimibe, which can effectively inhibit atherosclerosis by decreasing the macrophage index (Andrade-Neto et al., 2016). Meanwhile, it has been found recently that pyroptosis can be observed in plaques (Liu et al., 2023). Pyroptosis is accompanied by the release of large amounts of inflammatory factors, promoting plaque rupture and ultimately leading to cardiovascular events (Yarovinsky et al., 2023). Recent research has found that macrophages in plaques express abundant pyroptosis markers and are the main cells involved in pyroptosis, contributing to plaque instability (Wei et al., 2023). Our research shows that Clu can inhibit macrophage pyroptosis, which is the key mechanism by which Clu alleviates atherosclerosis and instability of plaques in diabetes.

This study has discovered a new target for treating diabetic atherosclerosis, which is a significant advancement in the field of cardiovascular research. However, it is important to acknowledge that this study has certain limitations that must be considered when interpreting the results. One notable limitation is that this study did not delve deeply into the specific signaling pathway by which CLU regulates the macrophage pyroptosis. Understanding these pathways could provide critical insights into how cellular processes are influenced and may lead to more effective therapeutic strategies. Recent study has indicated that GSDME activation can mediate the programmed cell death of macrophages, highlighting its role as an important molecular mechanism involved in both the formation and rupture of atherosclerotic plaques (Wei et al., 2023). Atherosclerosis remains one of the leading causes of cardiovascular diseases globally, making it essential to explore all potential mechanisms contributing to its progression (Buldak, 2022). Furthermore, we suspect that CLU may regulate macrophage pyroptosis by activing GSDME. To build upon these initial findings, we will conduct further research aimed at verifying our hypothesis regarding CLU’s regulatory effects on GSDME activity and subsequent implications for macrophage pyroptosis. Future studies will focus on elucidating detailed molecular interactions and exploring potential therapeutic interventions targeting these pathways to mitigate diabetic atherosclerosis effectively.

In conclusion, this study provides fresh insight into the role of CLU and sheds further light on a novel mechanism explaining that CLU ameliorates diabetic atherosclerosis via suppressing macrophage pyroptosis. Therefore, CLU may be a promising therapeutic target for macrophage inflammation and atherosclerosis induced by diabetes.

## Data Availability

The original contributions presented in the study are included in the article/Supplementary Material, further inquiries can be directed to the corresponding authors.
