# Supplementary material for: Clusterin ameliorates diabetic atherosclerosis by suppressing macrophage pyroptosis and activation
Source: Front Pharmacol. 2025 Apr 23;16:1536132. doi: 10.3389/fphar.2025.1536132 (PMC12055819; doi:10.3389/fphar.2025.1536132)
Supplement: Supplementary file 1 [file DataSheet1.docx]

Supplementary Material

# Supplementary Figures and Tables

## Supplementary Figures

**
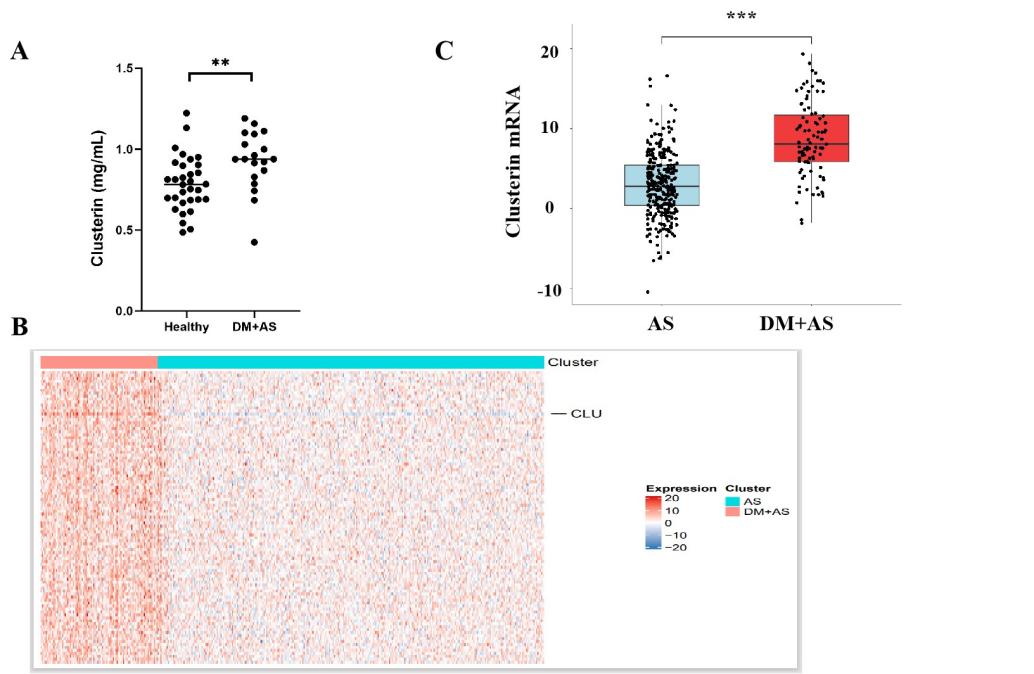
**

**Supplementary Figure 1.** **Increased CLU expression in patients with diabetes and** **atherosclerosis.** (A) The expression of CLU in diabetic atherosclerosis patients (n = 19) and healthy controls (n = 32) determined by ELISA. (B-C) The expressions of CLU in GSE221615. ^**^*P* < 0.01, ^***^*P* < 0.001. AS, Atherosclerosis; DM, Diabetes Mellitus.

## Supplementary Tables

**Supplementary Table 1. List of Primers Used for RT-qPCR**

| Genes | Forward primer (5'-3') | Reverse primer (5'-3') |
| --- | --- | --- |
| Human TNF-α | TGTAGCCCATGTTGTAGCAAACC | GAGGACCTGGGAGTAGATGAGGTA |
| Human IL-6 | TGGCTGAAAAAGATGGATGCT | TCTGCACAGCTCTGGCTTGT |
| Human IL-1β | CCACCTCCAGGGACAGGATA | AACACGCAGGACAGGTACAG |
| Human NLRP3 | GATCTTCGCTGCGATCAACAG | CGTGCATTATCTGAACCCCAC |
| Human GAPDH | CCACATCGCTCAGACACCAT | TTGACGGTGCCATGGAATTT |
| Murine TNF-α | CCTGTAGCCCACGTCGTAG | GGGAGTAGACAAGGTACAACCC |
| Murine IL-6 | GCTACCAAACTGGATATAATCAGGA | CCAGGTAGCTATGGTACTCCAGAA |
| Murine IL-1β | AGTTGACGGACCCCAAAAG | AGCTGGATGCTCTCATCAGG |
| Murine GAPDH | AAGAAGGTGGTGAAGCAGGCATC | CGGCATCGAAGGTGGAAGAGTG |

**Supplementary Table 2. Clinical characteristics.**

|  | Healthy controls (n = 32) | DM+AS (n = 19) |
| --- | --- | --- |
| Age (y) | 62.13 ± 1.77 | 63.63 ± 1.98 |
| Sex (male/female) | 22/10 | 13/6 |
| TC (mmol/L) | 4.55 ± 0.17 | 3.37 ± 0.19^***^ |
| HDL (mmol/L) | 1.34 ± 0.06 | 0.93 ± 0.04^***^ |
| LDL (mmol/L) | 2.45 (2.04-2.85) | 1.86 ± 0.18^***^ |
| TG (mmol/L) | 1.10 (0.84-1.70) | 1.61 ± 0.16 |
| FBG (mmol/L) | 4.92 ± 0.13 | 5.31 (4.63-7.01) |

DM + AS: patients with diabetes and atherosclerosis. ^***^*P* < 0.001.
